# Effects of the mGlu2/3 receptor agonist LY379268 on two models of disturbed auditory evoked brain oscillations in mice

**DOI:** 10.1038/s41398-023-02455-w

**Published:** 2023-05-06

**Authors:** Oana-Daniela Dormann, Niklas Schuelert, Holger Rosenbrock

**Affiliations:** Central Nervous System Diseases Research, Boehringer Ingelheim Pharma GmbH & Co. KG, Biberach Riss, Germany

**Keywords:** Predictive markers, Diagnostic markers, Diagnostic markers

## Abstract

Cognitive impairment is a core feature of schizophrenia and is poorly addressed by currently available medication. This is partly because the underlying circuits are insufficiently understood, and available animal models for brain dysfunction do not adequately mimic human pathology. To improve the translatability of animal studies and complement behavioral data, EEG measurements are being increasingly used in preclinical research. Brain oscillations are similar across species and can be impaired via several means. In this study, we used two approaches to impair early sensory processing and cortical oscillations in mice: a pharmacological model targeting NMDA receptor function in the whole brain via systemic MK-801 application and an optogenetic model targeting parvalbumin-positive (PV+) interneurons locally in the medial prefrontal cortex (mPFC). We evoked brain activity using auditory stimulation, a tool with high translatability from mouse to human. We then investigated the effect of LY379268, an agonist of mGlu2/3 receptors, a potential therapeutic target for schizophrenia, on single neuron and EEG responses. LY379268 was able to rescue MK-801-induced deficits for a variety of clinically relevant early sensory EEG biomarkers. Single neuron recordings revealed a strong effect of LY379268 on the signal-to-noise ratio during auditory stimulation and optogenetic inhibition of PV+ interneurons. Our results contribute to a better understanding of how group II metabotropic glutamate receptors modulate neuronal population and network activity under sensory stimulation while challenged pharmacologically or optogenetically.

## Introduction

Deficits of neural network function in the cortex are believed to be part of the pathophysiology of schizophrenia and are currently refractory to treatment [1]. Therefore, there is a high clinical need to develop treatments that target network dysfunction, for example, the excitatory–inhibitory (E/I) balance involving mainly the glutamatergic and GABAergic systems [2, 3]. Group II metabotropic glutamate receptors (mGluR2/3) appear to be a promising target in this regard. By acting as presynaptic auto-receptors, mGluR2/3 lower glutamate release. Several compounds that positively modulate the activity of mGluR2/3 have shown antipsychotic-like effects in animal models [4, 5] and in a clinical phase II trial in patients with schizophrenia [6]. However, these results could not be confirmed in subsequent clinical trials [7–10]. Some of the reasons for these discrepancies could be: (i) the post-synaptic action of the mGluR2/3, which can produce an opposing increase in glutamatergic transmission [11], (ii) inadequate dosing [12], (iii) improper patient population selection, or (iv) a higher than expected placebo effect [13]. Nonetheless, mGluR2/3 activation was shown to reverse working memory deficits and BOLD (blood oxygenation level-dependent) signal induced by the NMDA receptor antagonist ketamine in healthy subjects [14, 15], supporting the NMDA receptor hypofunction hypothesis of schizophrenia [16–18]. This hypothesis has its foundation in many functional and histological studies in patients, which showed that specific brain regions like the dorsolateral prefrontal cortex (DLPFC) are particularly affected and exhibit changes in synapse number and interneuron marker expression [19–22]. Such regional specificity is not reflected in pharmacological models of schizophrenia symptoms, like systemic NMDA receptor antagonism. These models can, however, achieve target selectivity by tweaking the dosage of the antagonist, i.e., at a low dose, their action is mediated mainly by inhibitory parvalbumin-expressing (PV) interneurons [23, 24]. Nevertheless, complementary approaches are needed for specific manipulation of brain circuits suspected to be more strongly involved in cortical network dysfunction in schizophrenia.

To achieve this goal, in this study, we used two approaches to model interneuron hypofunction: (i) systemic application of the NMDA receptor antagonist MK-801 and (ii) targeted optogenetic inhibition of PV interneurons in the medial prefrontal cortex (mPFC) of mice. Optogenetics enables the expression of light-sensitive proteins (initially found in algae and subsequently engineered in the lab for improved kinetics and light sensitivity) via modified, non-replicating viruses to manipulate neuronal activity [25]. These viruses can be engineered to ensure cell specificity (through specific promoters or viral capsids or by using the Cre-lox system). Here we used a mouse line expressing Cre recombinase in PV interneurons [26] and expressed the light activatable proton pump Archaeorhodopsin (Arch) in the mPFC.

The first model was assessed via supradural screws, to allow the recording of cortical oscillations, and the second via intra-laminar tetrodes (4-wire electrodes), facilitating oscillation and extracellular neuron activity detection. We used auditory stimulation to evoke network oscillations, a paradigm with very good translatability from mouse to human and shown to reveal network dysfunction in schizophrenia [22–26]. These paradigms were used to assess the ability of the mGluR2/3 agonist LY379268 to reverse deficits induced by MK-801 or targeted optogenetic inhibition. Our results contribute to the further understanding of the mechanism of action and potential of mGluR2/3 activation on the modulation of the E/I balance.

## Methods

### Animals

For the pharmacological experiments, 19 C57Bl/6 male mice weighing 25–28 g upon arrival, provided by Janvier (France), were used. For the optogenetic experiments, 8 PV-Cre mice male mice (B6;129P2Pvalbtm1(cre)Arbr/J) aged 12–14 weeks were used. After surgery, animals were singly housed to avoid damage to the electrodes, with free access to food and water. All experimental procedures were authorized by the Local Animal Care and Use Committee in accordance with local animal care guidelines, AAALAC regulations, and the USDA Animal Welfare Act.

### Procedures applied to the mice recorded with supradural screws

#### Surgery

The procedure was described in detail in a previous study [27]. Briefly, wild-type mice were placed under isoflurane anesthesia on a heating pad. Gold-plated electrode screws attached to gold pins were implanted into the skull under sterile conditions at the following coordinates: auditory cortex AP −2.7 mm, ML ±4 mm, PFC AP 1.8 mm, ML ±1 mm, measured from bregma. The reference electrode was placed 1.5 mm posterior to lambda. The gold pins were secured in place using dental cement. Postsurgical analgesia was provided for 3 days (Metacam 0.1 mg/kg s.c.), and recovery was allowed for 1 week.

#### Auditory stimulation

Animals were first habituated to the sound-attenuated chambers for several days (Med Associates Inc.). Auditory stimulation was performed using an audio generator, and electrical signals were recorded with a Neurologger and analyzed offline. Within a recording session, the ERP protocol (300 repetitions of white noise click pairs, spaced 8 s apart) or ASSR protocol (300 repetitions of 2-s-long click trains consisting of 80 white noise clicks, spaced 10 s apart, at 85 dB) were presented. All mice received all treatments in a randomized cross-over design.

#### Data analysis

Data were analyzed using Analyzer2 (Brain Vision) as described previously in detail [27] and further processed using custom-made scripts in R.

### Procedures applied to the optogenetically manipulated mice

#### Surgery

The procedure was described in detail in a previous study [28]. Briefly, PV-Cre mice were placed under isoflurane anesthesia on a heating pad. Four stainless steel screws were used as anchors (two of which above the cerebellum also served as reference). 200 nl virus (AAV5_ss_EF1a-DIO-eYFP, titer 1.09E + 13 VG/mL) were injected into the PFC bilaterally, at AP: 1.75, ML: ±0.4, DV: −2.75, measured from bregma. Microdrives holding 4 independently movable tetrodes and 2 optic fibers were implanted directly above the injected area and secured to the skull using dental cement.

#### Auditory stimulation and optogenetic inhibition

The animals were allowed to recover and express the virus, then were subjected to a battery of tests we described in a previous publication [28] before the mGluR2/3 agonist LY379268 was tested. Recording sessions included 600 white-noise click trains at 40 Hz, 10 s apart, of which 50% were delivered during optogenetic inhibition of PV+ interneurons in a pseudorandom sequence. All mice received both treatments (saline and LY379268) in a cross-over design, with at least 3 days between the two recording sessions. The data was filtered and thresholded online for spike detection, and neuronal spikes were saved on disk at a sampling rate of 40 kHz, while the continuous LFP was saved and sampled at 1 kHz.

#### Data analysis

Spike clustering was performed semiautomatically offline using Matlab (Code availability under https://github.com/adredish/MClust-Spike-Sorting-Toolbox), the LFP was processed using Analyzer2 and all data were pulled together for the final analyses using custom scripts in R [28].

#### Histology

At the end of the experiment, the mice were perfused intracardially with ice-cold PBS followed by 4% PFA. The brains were cut on a vibratome in 50 µm sections and mounted on glass slides to check for virus expression and electrode position. We reported on the histology in our previously published study since there we used the same animals we used in the current study [28].

#### Statistical analysis

Statistical analysis was performed using MATLAB (MathWorks, for firing rate analysis), SAS (SAS Institute, Cary, NC, USA, for ERP and ASSR parameters), and GraphPad Prism.

## Results

### The mGluR2/3 agonist LY379268 restored MK801-induced AERP deficits and abnormal gamma oscillations

**Fig. 1:**
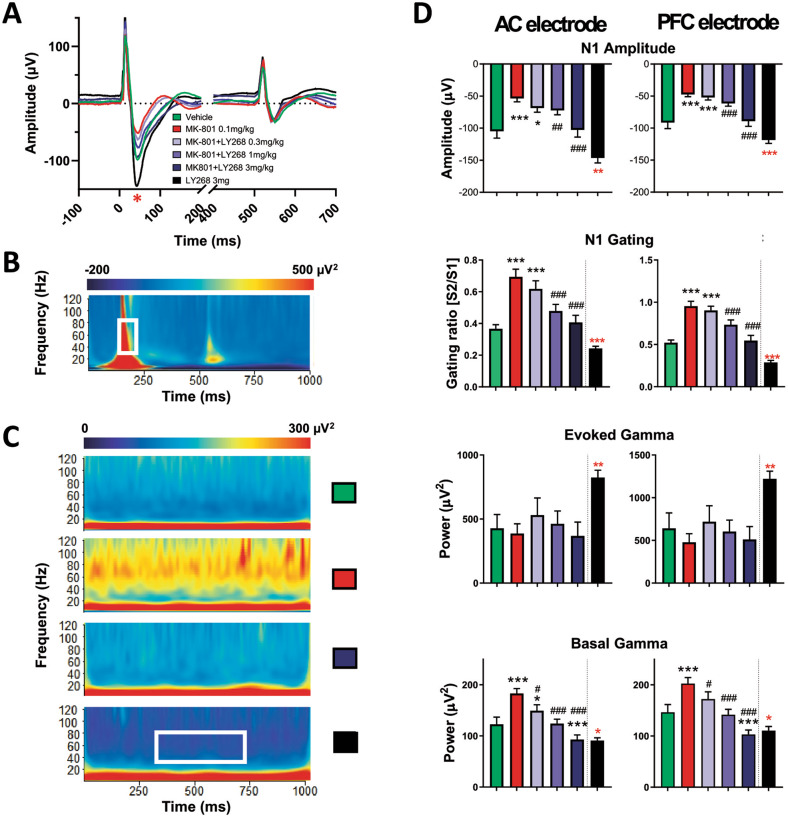
AERP analysis for the auditory cortex and the medial prefrontal cortex. **A** AERP average traces for all mice for the AC, highlighting the incremental rescue effect with increasing compound concentration. The red asterisk indicates n1 for the first click. **B** Representative example of Morlet wavelet transformation for evoked oscillations. The white rectangle indicates the area used to calculate evoked gamma power. **C** Representative examples of Morlet wavelet transformation for baseline oscillations for vehicle, MK-801, MK-801 + 3 mg/kg LY379268, and 3 mg/kg LY379268 in one mouse. The white rectangle indicates the area used to calculate average basal oscillations per mouse per condition. **D** Parameters are calculated based on the AERP and the Morlet wavelet transformation. Least squares means model, bar graphs depict mean ± SEM, *p < 0.05, **p < 0.01, ***p < 0.001 compared to vehicle; ^#^*p* < 0.05, ^##^*p* < 0.01, ^###^*p* < 0.001 compared to MK-801 treatment, *n* = 19 mice. The dashed line between the dark blue and the black bar indicates that the compound alone was administered in an additional round of experiments and was not included in the initial cross-over design.

For the auditory evoked related potentials (AERP), the double-click paradigm was used to assess whether LY379268 can rescue the deficits induced by the non-competitive NMDA receptor antagonist MK-801 (namely reduced n1 amplitude, reduced gating, and increased gamma oscillation power, Fig. 1). Such changes indicate impaired sensory processing and gating (i.e., the inability to filter out distracting or background stimuli). Here we used 0.1 mg/kg MK-801 for the following reasons: (i) it produces EEG deficits without motor impairment or hyperactivity [27], and (ii) low MK-801 doses mainly affect interneurons and elicit network disinhibition [23, 24]. We detected a dose-dependent rescue of the deficits induced by MK-801 on n1 amplitude, n1 gating, and basal gamma oscillations (30–80 Hz) through the application of the mGluR2/3 agonist LY379298 (Fig. 1D). We could show this both in the auditory cortex (AC) and in the mPFC [23]. The agonist alone increased evoked gamma power, unlike a recent study reporting no effect on this parameter [29].

### LY379268 restored the MK801-induced ASSR 40 Hz coherence deficit

**Fig. 2:**
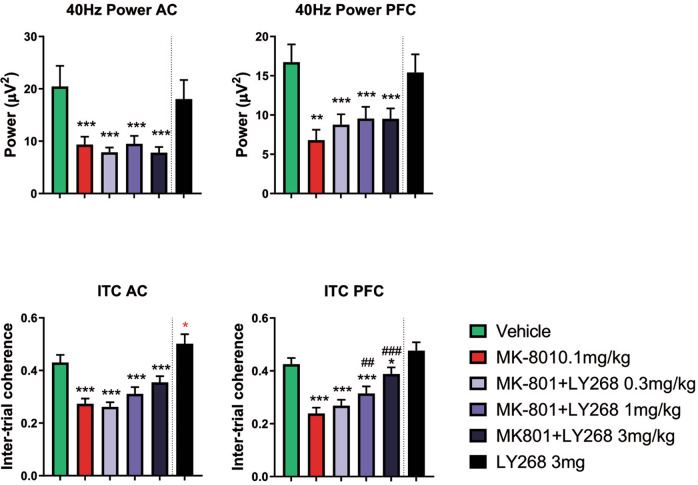
Auditory steady-state characteristics under MK-801 challenge and LY379268 treatment. Least squares means model, bar graphs depict mean ± SEM, *p < 0.05, **p < 0.01, ***p < 0.001 compared to vehicle; ^##^*p* < 0.01, ^###^*p* < 0.001 compared to MK-801 treatment. The dashed line between the dark blue and the black bar indicates that the compound alone was administered in an additional round of experiments and was not included in the initial cross-over design.

The next paradigm we investigated was the auditory steady-state response (ASSR) at 40 Hz. This type of auditory stimulation tests the capacity of a neuronal circuit to sustain oscillations in the gamma band and is specifically impaired in patients with schizophrenia. MK-801 induced a strong reduction in power at 40 Hz and inter-trial coherence (ITC). Co-treatment with LY379268 did not rescue the MK-801-induced reduction in power at 40 Hz but could dose-dependently rescue the ITC deficits in the mPFC, but not in the AC (Fig. 2). Interestingly, we detected an increase in ITC in the AC when administering the compound alone at 3 mg/kg.

### LY379268 reduced background neuronal activity and increased the initial response to auditory stimuli in the mPFC

**Fig. 3:**
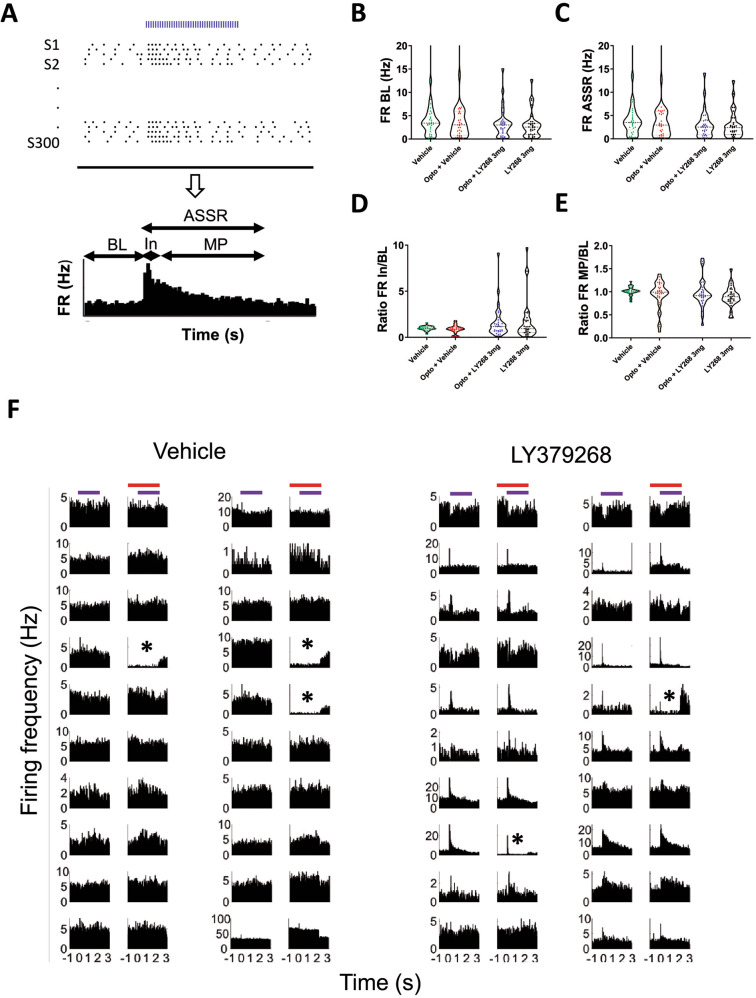
Firing rate pattern of neurons during auditory steady-state response. **A** Theoretical approach to obtaining a peristimulus histogram (PSTH) for a neuron. The timestamps of individual spikes were aligned to the stimulus (Sn) start (the ASSR, blue dashed bar), pooled together, grouped into time bins (20 ms), and divided by time to obtain the firing rate in Hz. **B** Firing rate during baseline of all recorded neurons. **C** Firing rate during ASSR. **D** Ratio of the firing rate during the initial response and baseline firing rate. **E** Ratio of the firing rate during the maintenance phase and baseline. **F** Examples PSTHs. Each pair of neighboring PSTHs belongs to one neuron during ASSR (blue bar) without and with optogenetic inhibition of PV+ interneurons (blue and red bar). Asterisk-marked PSTHs belong to putative PV interneurons. Vehicle *n* = 33, LY379269 *n* = 31. BL baseline, In initial phase, MP maintenance phase.

We hypothesized that PV interneurons are critical for regulating gamma-band oscillations and therefore implemented a model to selectively silence these neurons in the mPFC. We extensively described this approach in a previous study [28]. Within one session, we had 50% of the trials under normal PV+ IN function and 50% of the trials under optogenetic PV+ IN inhibition, intertwined in random sequence, either after the vehicle (green and red on the graphs in Fig. 3) or after LY379268 3 mg/kg application (blue and black).

The violin plots give an overview of firing rate distributions for all recorded neurons before and during ASSR stimulation in the four different conditions. Given the neuronal composition of the cortex, most recorded neurons (80–90%) were excitatory, and only a minority were inhibitory. Although we did not have enough data to perform statistical analysis, we could corroborate current data with data from our previously published study [28] and infer that also in this population of recorded neurons, a few showed decreased firing rate due to optogenetic inhibition (the PV+ interneurons) and a larger proportion showed increased firing rate due to disinhibition. Here we defined a set of time intervals that we named baseline (BL, 2 s up to the start of auditory stimulation), initial response (In, the first 50 ms of the response to auditory stimulation), and maintenance phase (MP, 50–2000 ms into the steady-state response, intervals adapted based on data from humans [30]), which we pooled for all 300 click trains per neuron, per condition (Fig. 3A). We observed that the inhibition of PV+ interneurons widened the distribution of firing rates, since the firing of inhibited PV+ neurons went down, while other neurons were disinhibited and fired more, both during BL and ASSR (Fig. 3B, C). The mGluR2/3 agonist-induced an opposite trend, therefore tightening the distribution of firing rates (Fig. 3B, C). Furthermore, we could observe a striking effect on the initial response to auditory stimulation. LY379268 strongly increased initial firing in some neurons while decreasing it in others (Fig. 3D, F). One PV+ interneuron, whose firing rate was essentially abolished by optogenetic inhibition, showed a strong initial response during ASSR and treatment with the mGluR2/3 agonist (see Fig. 3F, LY379268 column, asterisk, third row from the bottom). We could not identify such strong responses in mPFC neurons during vehicle treatment (see Fig. 3F for examples). We did not perform extensive analysis to assign a cell identity to recorded neurons (i.e., putative pyramidal vs putative PV+). However, neurons that showed reduced firing rate during optogenetics were most probably PV+ interneurons (see asterisk marked neurons in Fig. 3F).

### LY379268 reverted oscillation deficits induced by optogenetic inhibition of mPFC PV+ interneurons during ASSR

**Fig. 4:**
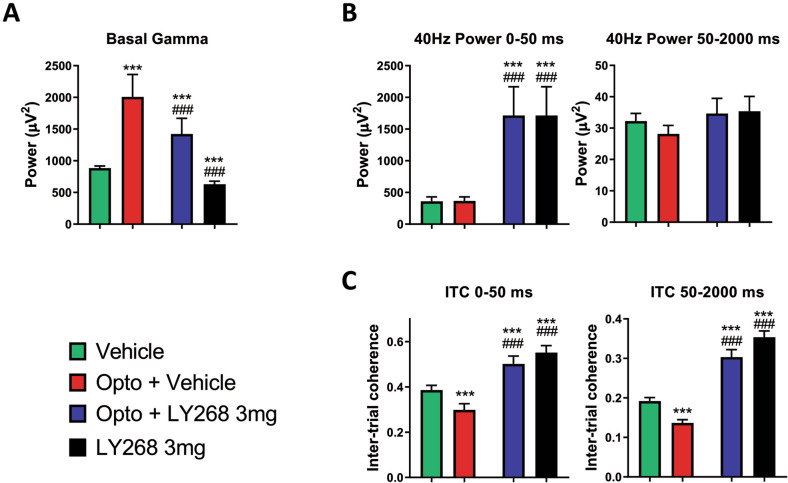
Auditory steady-state response parameters during optogenetic inhibition of PV+ interneurons and LY379268 treatment. Least squares means model, bar graphs depict mean + SEM, ***p < 0.001 compared to vehicle (in green); ^###^*p* < 0.001 compared to opto + vehicle (in red).

As previously reported [28], inhibition of PV+ INs strongly increased basal gamma oscillations. The mGluR2/3 agonist could counterbalance the increase in basal gamma oscillations to a significant degree and, on its own, led to decreased basal oscillations below control levels (Fig. 4A).

As in the single neuron analysis, we split the analysis of ASSR power and ITC into two phases, the initial and maintenance phase [30]. The optogenetic manipulation induced a trend toward decreased power during the maintenance phase (*p* = 0.06, *n* = 25 electrodes/8 mice) and had no effect on the initial phase. The compound, however, had a very strong effect on power at 40 Hz in the initial phase but not in the maintenance phase, therefore reflecting single neuron activity changes (Fig. 4B). Interestingly, the changes in ITC were independent of the phase, showing a significant decrease due to PV+ IN inhibition and an increase through the compound application (Fig. 4C). A two-way ANOVA test on each of the above-mentioned parameters revealed no interaction between the pharmacological and the optogenetic manipulation.

## Discussion

To our knowledge, this study is the first to assess the effects of LY379268 on network function in two models of E/I imbalance. The nature of the deficits and reversal by this compound bring mechanistic insights to both the induced deficits and the role of mGluR2/3 in these deficits. The fact that the compound restored EEG deficits induced by brain-wide increased E/I ratio via systemic administration of the NMDA receptor antagonist MK-801 suggests that it reduces excitation to restore neuronal function. However, in the second model, the compound partially restored baseline oscillation power while driving the ITC and initial power at 40 Hz above vehicle conditions, highlighting that a whole-brain activation of these receptors is detrimental in the absence of a whole-brain impairment.

### Relevance of pharmacological models for the study of E/I balance

Several studies of auditory steady-state response in patients with schizophrenia have described reduced power and phase-locking at stimulation frequencies around 40 Hz [31–35]. These deficits are indicators of a disturbed E/I balance and constitute emergent biomarkers for psychiatric disorders [36].

In preclinical studies, an E/I imbalance can be modeled by the systemic application of NMDA receptor antagonists such as ketamine, PCP, or MK-801 [16]. These drugs induce sensory processing deficits like those measured in patients. Recent studies indicated that Group II receptor agonists exert restorative effects on impaired auditory processing [37, 38] while attenuating deficits in some but not all behavioral tasks [39–42].

In this study, the mGluR2/3 agonist LY379268 restored all MK-801-induced deficits in AERP and 40 Hz ASSR, with the notable exception of power at 40 Hz. This restoration is in line with clinical studies, where Group II receptor agonists reversed the effects of an NMDA receptor antagonist (ketamine) detected using fMRI-BOLD [14, 15]. Thus, our results add to the plethora of preclinical studies supporting the beneficial role of mGluR2/3 activation for the restoration of neuronal E/I balance.

### Lessons from an optogenetic model

Since the ultimate goal is to improve cognitive deficits in patients and cognition is heavily dependent on the prefrontal cortex (PFC) among other regions, here we used a model in which PFC function was selectively impaired. Specifically, we targeted the function of an interneuron subclass that is strongly affected in patients with schizophrenia and is of great relevance to neuronal network function and the E/I balance [21, 43–46]. We described this model in detail in a previous publication [28] and showed that gamma power is correlated to the firing of putative pyramidal neurons and that ITC is decreased during 40 Hz ASSR when PV+ interneuron function is decreased in the mPFC of mice. Here we additionally demonstrate that the mGluR2/3 agonist LY379268 could partially restore basal gamma deficits while inducing an ‘overshoot’ in ITC and initial power at 40 Hz, unlike the restoration towards baseline we observed in the MK-801 model.

These data nicely illustrate differences between brain-wide and circuit-specific pre-clinical models. Interestingly, we could detect a dissociation between power at 40 Hz and ITC: in the pharmacological model, power at 40 Hz was reduced and not affected by LY379268, while ITC at 40 Hz was reduced and was rescued by LY379268; in the optogenetic model, the power at 40 Hz was not affected by PV silencing, while ITC at 40 Hz was reduced, and both parameters were increased by LY379268. These results may reflect the different sources of activity patterns (local versus distant), which we discuss in the next section.

### Limitations and outlook

One drawback of our studies is that none of the setups enables us to determine the sources of these patterns of activity. However, after comparing the two models, we are tempted to speculate that ITC and gamma oscillations are phenomena that rely more on local network dynamics and particularly on PV+ INs, while power at 40 Hz is regulated by incoming inputs (e.g., from the auditory cortex or thalamus). These two measurements could be valuable for pinpointing affected brain regions and stratifying patients. In line with these ideas, the strength of 40 Hz oscillation power impairment has been recently shown to be predictive of clinical outcomes for patients at risk for psychosis [47] and indicative of disease progression [48]. Additionally, a recent exploratory analysis of clinical trial data revealed drug effectiveness in early- versus late-in-disease patients (with the limitation that the study was not designed to specifically test this) [13]. These results are in line with the hypothesis that some patients display a *hyper*-glutamatergic state early on, which develops into a *hypo*-glutamatergic as the disease becomes chronic [49]. This might mean that only early intervention with glutamate-decreasing compounds would show a clear benefit for the patient. Such data demonstrate that clinical research is well on its way to precision psychiatry and that refining physiological biomarkers for the diagnosis and prognosis of psychiatric disorders could one day dramatically improve patient selection and treatment.

### Closing remarks

In the present study, we showed that the deficits identified in two models of acute E/I imbalance were differentially reverted by the mGluR2/3 agonist LY379268, potentially portraying different patient populations. Identifying patient-specific deficits will be the key to clinical trial inclusion criteria in the spirit of precision psychiatry and to increasing clinical trial success in psychiatric disorders. Our data strengthen the idea that Group II metabotropic glutamate receptors are worth pursuing as a potential therapeutic target for specific patient subpopulations, and therefore efforts to optimize drug testing and refine biomarkers with the aid of EEG/MEG/fMRI should be continued.
